# Vascular tumors in infants and adolescents

**DOI:** 10.1186/s13244-019-0718-6

**Published:** 2019-03-13

**Authors:** Moritz Wildgruber, Maliha Sadick, René Müller-Wille, Walter A. Wohlgemuth

**Affiliations:** 1 Institut für Klinische Radiologie, Universitätsklinikum Münster, Albert-Schweitzer Campus 1, 48149 Münster, Germany; 2 Institut für Klinische Radiologie und Nuklearmedizin, Universitätsmedizin Mannheim, Theodor-Kutzer-Ufer 1-3, 68167 Mannheim, Germany; 3 Institut für Diagnostische und Interventionelle Radiologie, Universitätsmedizin Göttingen, Robert-Koch Strasse 40, 37075 Göttingen, Germany; 4 Universitätsklinik und Poliklinik für Radiologie, Ernst-Grube-Strasse 40, 06120 Halle (Saale), Germany

**Keywords:** Vascular anomalies, Vascular tumor, Hemangioma, Imaging

## Abstract

Malignant vascular tumors as part of the vascular anomalies spectrum are extremely rare in children and young adults. Instead, benign vascular neoplasias are frequently encountered in the pediatric patient population. While vascular malformations are congenital vascular lesions, originating from a mesenchymal stem cell defect, vascular tumors are neoplastic transformations of endothelial and other vascular cells. The appropriate differential diagnosis and nomenclature according to the classification of the International Society for the Study of Vascular Anomalies (ISSVA) is decisive to initiate correct therapy. While infantile hemangioma can be routinely diagnosed by clinical means and rarely require therapy, more rare vascular tumors are frequently difficult to diagnose, require dedicated cross-sectional imaging, and benefit from an interdisciplinary treatment approach. The focus of this review is to provide an overview over the spectrum of vascular tumors, typical imaging characteristics, and summarize treatment options including interventional radiology approaches.

## Key points

- Vascular tumors are distinct from vascular malformations and should be appropriately classified according to the classification of the International Society for the Study of Vascular Anomalies (ISSVA); misdiagnosis and inappropriate nomenclature is frequent.
- Infantile hemangiomas are the most frequent vascular tumors in children and most frequently do not require treatment.
- Locally aggressive as well as borderline tumors can present with typical imaging appearance depending on their degree of vascularity. Complications of benign vascular tumors include ulceration and bleeding which require immediate therapy, consisting of medical, surgical, and interventional radiology approaches.
- Minimally invasive image-guided treatment strategies can be used for palliation and to manage complications.

## Introduction

Vascular tumors comprise a vast spectrum of diseases and are therefore difficult to diagnose and classify. Benign vascular tumors can be mistaken for vascular malformations, but even more frequently vascular malformations are misdiagnosed as vascular tumors, such as infantile hemangiomas. Inappropriate misnomers and delayed diagnoses as well as false classification are responsible for wrong treatment approaches, which may delay appropriate therapy, or lead to significant morbidity and mortality. Herein, we discuss the differential diagnosis of vascular tumors from vascular malformations when appropriate; for an overview of the large spectrum of vascular malformations, we refer to recently published work [1–4].

Vascular tumors are characterized by a different pathobiology compared to vascular malformations. Vascular tumors are neoplastic, characterized by increased proliferation rates of endothelial and other vascular cells [5]. Instead, vascular malformations are congenital vascular lesions, grow commensurately with the child, cannot involute, and have abilities to expand hemodynamically. Vascular malformations demonstrate no neoplastic endothelial cell proliferation, contain small and large vascular channels lined by flat endothelium, have a unilamellar basement membrane, and have normal mast cell counts [1, 6–8].

Infantile hemangiomas initiate a rapid growth during the first months of life and regress spontaneously later on [9]. Congenital hemangiomas, much less frequent than infantile hemangiomas, can be divided into rapidly involuting congenital hemangioma (RICH), partially involuting congenital hemangiomas (PICH), and non-involuting congenital hemangiomas (NICH) due to their biological behavior after birth [10]. Most importantly, infantile hemangiomas rarely require imaging for correct diagnosis and similarly important, infantile hemangiomas rarely require aggressive treatment. Malignant vascular tumors instead exhibit a high metastatic potential similarly as in adults and thus require rapid diagnosis and therapy. In between benign and malignant vascular tumors, there is a specific group of tumors with locally aggressive behavior and potential risk of life-threatening coagulation disorders such as Kasabach-Merrit phenomenon seen in Tufted Angioma and Kaposiform Hemangioendothelioma [11]. These tumors are probably the most challenging to treat, and due to their rareness, no evidence-based guidelines and treatment strategies exist.

The aim of this review is to summarize characteristics of vascular tumors as part of the vascular anomalies spectrum in children and young adults and to provide examples of potential treatment approaches embedded in an interdisciplinary setting of pediatric oncologists, radiologists, cardiologists, surgeons, pathologists, hemostasiologists, and others, that are required to provide both rapid and definite diagnosis for adequate treatment. After some general remarks on the classification of vascular tumors and the diagnostic imaging workup, we present the most important vascular tumors in the order of the ISSVA classification and describe epidemiology, etiology, pathology, clinical features typical presentation on radiologic imaging, therapeutic concepts, as well as clinical complications of each entity.

## Classification of vascular tumors

Most importantly, vascular tumors have to be distinguished from vascular malformations based on the classification system of the International Society for the Study of Vascular Anomalies ISSVA, last updated in 2018 (http://www.issva.org/UserFiles/file/ISSVA-Classification-2018.pdf). In 2013, the World Health Organization (WHO) updated their classification of vascular soft tissue tumors. In the current version, the terminology was mostly left unchanged and pediatric tumors where not classified independently from adult tumors. However, the intermediate malignant category was divided into locally aggressive and rarely metastasizing tumors. Especially with respect to the benign vascular tumors, the WHO classification still relies on outdated pathological terminology, such as ‘cavernous’ and ‘capillary’ hemangioma. It also does not differentiate between vascular tumors and vascular malformations, which has to be avoided for the sake of appropriate therapy. In contrast, the ISSVA classification respects the indispensable differentiation between vascular tumors and vascular malformations and represents a very appropriate scheme to stratify proliferative vascular lesions (Table 1). These different classification systems (old pathological nomenclature of WHO and updated clinical-biological classification of ISSVA) may lead to difficulties in interpreting these disease findings. The ISSVA classification should be the clinical gold standard for patient care today.

**Table 1:** ISSVA classification of vascular tumors

Benign vascular tumors	
Infantile hemangioma	
Congenital hemangioma	
• Rapidly involuting (RICH)^a^	
• Non-involuting (NICH)	
• Partially involuting (PICH)	
Tufted angioma^a^	
Spindle-cell hemangioma	
Epitheloid hemangioma	
Pyogenic granuloma	
Others	
Locally aggressive or borderline vascular tumors	
Kaposiform hemangioendothelioma^a^	
Retiform hemangioendothelioma	
Papillary intralymphatic angioendothelioma (PILA), Dabska tumor	
Composite hemangioendothelioma	
Pseudomyogenic hemangioendothelioma	
Polymorpous hemangioendothelioma	
Hemangioendothelioma not otherwise specified	
Kaposi sarcoma	
Others	
Malignant vascular tumors	
Angiosarcoma	
Epitheloid hemangioendothelioma	
Others	

Of note: (1) reactive proliferative vascular lesions are listed within benign tumors, and (2) tufted angioma and Kaposiform hemangioendothelioma are frequently regarded as part of a spectrum rather distinct entities

^a^Lesions may be associated with thrombocytopenia and/or consumptive coagulopathy (Kasabach-Merrit phenomenon)

## Diagnostic imaging workup

The need for diagnostic and especially cross-sectional imaging increases with the degree of malignancy of vascular tumors. While infantile hemangiomas rarely require imaging to establish the diagnosis or to monitor disease and therapy effects, borderline as well as malignant tumors always request imaging to assess the extent of disease, the involvement and penetration of different tissues, as well as potential metastasis [12]. A brief overview on the most important clinical features, pathological markers, and recommended imaging tools is given in Table 2.

**Table 2:** Overview of the characteristics features and need for imaging of vascular tumors

Entity	Pathology	Clinical presentation	Imaging
Infantile hemangioma	GLUT-1 positive	Solid vascularized mass, cutaneous lesions present frequently as raspberry-like patches at any part of the body, lesion typically do not appear before 2 weeks of age	Rarely required, if required US most frequently sufficient
Congenital hemangioma	GLUT-1 negative	Similar appearance compared to infantile hemangioma, present at birth	Rarely required, if required US most frequently sufficient
Pyogenic granuloma	GLUT-1 negative	Small (≤ 1 cm), sessile or pedunculated red papule or nodule	Not required
TA/kaposiform hemangioendothelioma	Positive for PROX-1, Podoplanin/D2–40, LYVE-1, CD31, and CD34 but GLUT-1 negative	Expanding ecchymotic firm mass with purpura and accompanying lymphedema	MRI including MR angiography recommended
PILA/Dapska tumor	Peri- and intravascular lymphocytic infiltrates are common, endothelials cells frequently positive for CD31, CD34, ‘D2–40 and VEGFR-3	Red infiltrating singular plaque, affecting cutis and subcutis	Not required
Kaposi sarcoma	Immunoreactivity for LANA-1, an HHV-8 viral antigen is pathognomonic	Sharply demarcated patch or later plaque-like infiltrates of skin and subcutaneous tissue (cutaneous manifestations)	Imaging rarely required for cutaneous manifestation, recommended for visceral manifestations
Epitheloid hemangioendothelioma	Positive for CD31 and factor VIII, variably for CD34, epitheloid endothelial cells within a hyalinized or myxoid stroma	Red-brownish plaque or nodule (cutaneous lesions)	MRI including MR angiography recommended, CT imaging for stating purpose
Angiosarcoma	Positive for CD31 and CD34, and also for factor VIII, necrosis and hemorrhage common, angiosarcomas can be GLUT-1 positive	Diffuse infiltrating mass, clinical findings rarely specific	MRI including MR angiography recommended, CT imaging for stating purpose, PET imaging may be helpful in dedicated cases

Ultrasound (US) is the modality of choice as it allows to assess both the extension of the disease in children as well as the degree of vascularity and associated Doppler spectra. Especially if there is ambiguity in determining if the lesion is a hemangioma/soft tissue mass or a vascular/lymphatic malformation, performing US can clearly discriminate if the lesion in question is cystic (rather malformation) or solid (rather hemangioma). In general, computed tomography (CT) should be avoided in children and adolescents due to increased susceptibility to ionizing radiation. Only in cases of osseous involvement of vascular tumors especially in the head-and-neck region CT may be applied to assess the degree of bone involvement and destruction. Magnetic resonance imaging (MRI) is the modality of choice to assess disease extent and differential diagnosis especially in deep tissues that cannot be diagnosed clinically. A summary of typical MR imaging features for vascular tumors as well as imaging differential diagnosis are provided in Table 3. Of note, MR imaging in small children requires general anesthesia. A suggested, MR imaging protocol is presented in Table 4. Positron emission tomography (PET/PET-CT) due to the associated ionizing radiation is reserved for tumors suspicious of a high degree of malignancy and assessment of metastasis.

**Table 3:** MR imaging of vascular tumors of different malignancy

Vascular tumors				
	Hemangioma (infantile/congenital)	Tufted angioma/kaposiform hemangioendothelioma	Epitheloid hemangioendothelioma	Angiosarcoma
MRI morphology	Solid, frequently homogeneous well-defined mass	Diffuse infiltrating mass permeating all soft tissue structures	Solid mass with ill-defined margins	Diffuse inhomogeneous mass, infiltrating all tissue types
MRI signal				
T1 pre-contrast	Isointense to muscle	Isointense to muscle	Isointense to muscle	Isointense to muscle
T2 (to be performed with fat saturation)	Hyperintense to muscle	Hyperintense to muscle, septal architecture perpendicular to the skin, often with edema	Moderate hyperintense to muscle	Hyperintense to muscle
Fat-saturated T1 post-contrast	Hyperintense to muscle and flow-voids	Hyperintense to muscle, septal enhancement	Moderate hyperintense to muscle	Hyperintense to muscle (central necrosis frequent)
MRI flow-characteristics				
MR-angiography	Fast-flow, tumor blush	Fast flow, tumor blush	Slow flow	Slow-flow
Imaging differential diagnosis	Venous (in case of slow flow hemangioma) or arteriovenous malformation (in case of fast-flow), KHE, macular stains/capillary malformation, soft tissue sarcoma	Hemangioma, soft tissue sarcoma, extraosseus Ewing sarcoma, kaposiform lymphangiomatosis	Hemangioma (such as epitheloid hemangioma), HCC (in case of liver manifestation), lymphoma (in case of lymphatic manifestation), sarcoma	Can mimic any malignant highly vascularized tumor (breast, soft tissue, bone, visceral, head, and neck), intravascular angiosarcoma can resemble thrombosis or atheroma

**Table 4:** Example of MR imaging protocol

Sequence	Alternative
T1 SE	
STIR/TIRM (recommended in at least two planes)	T2 Dixon, PD with fat-saturation (in case of small field of view/region of interest)
Dynamic MR angiography (especially in case of suspected fast-flow)	
T1 with fat-saturation after gadolinium administration (recommended in two planes)	T1 Dixon after gadolinium administration

### Benign vascular tumors

#### Infantile hemangioma

##### Epidemiology

Infantile hemangiomas are the most frequent benign tumors of infancy, with a prevalence of 4–5%, a clear female predominance (~ 2.5 times higher), and increased frequency in Caucasian children [9]. Infantile hemangiomas are more likely to occur in cases of low birth weight and decreasing gestational age. In case of a birth weight less than 1000 g, the prevalence of infantile hemangiomas is as high as 23% [13].

##### Pathology

Infantile hemangiomas are based on dysregulation of both vasculogenesis and angiogenesis. Hypoxic stress seems to act as a major trigger, resulting in increased expression of proangiogenic factors such as VEGF [14, 15]. Stimulated by VEGF overexpression, mesenchymal stem cells differentiate into immature endothelial cells, pericytes and dendritic cells, as well as mesenchymal cells with adipogenic potential [16]. During the rapid early growth phase, these immature endothelial cells dominate, forming tumorous masses. Later on, during regression, the vascular lumina become narrower and the immature vasculature is continuously replaced by a fibrofatty residuum [7]. Because of the complex cellular nature of hemangiomas, the proliferative phase may continue as the involutive phase slowly begins to dominate. Throughout their growth, endothelial cells of infantile hemangiomas express the immunological marker glucose transporter one (GLUT-1), which is frequently applied to differentiate vascular tumors from malformations in tissue biopsies. However, the GLUT-1 transporter is still of debate: while frequently being regarded to be specific for infantile hemangioma, it is expressed similarly by other tumors [17]; recent publications have demonstrated positive GLUT-1 staining also in epitheloid hemangioendotheliomas, angiosarcomas, and angiokeratomas, whereas it was confirmed to be negative in most vascular malformations [18]. GLUT-1 is therefore considered to be helpful in differentiating vascular neoplasms from malformations in histological specimens, but positive GLUT-1 staining is not specific for infantile hemangiomas. Recently, increased expression of the programmed cell death 1 receptor (PD-1) has been demonstrated in both infantile hemangiomas and venous malformations and thus raises the possibility for immunotherapy using PD-1 ligands [19].

##### Clinical features

**Fig. 1:**
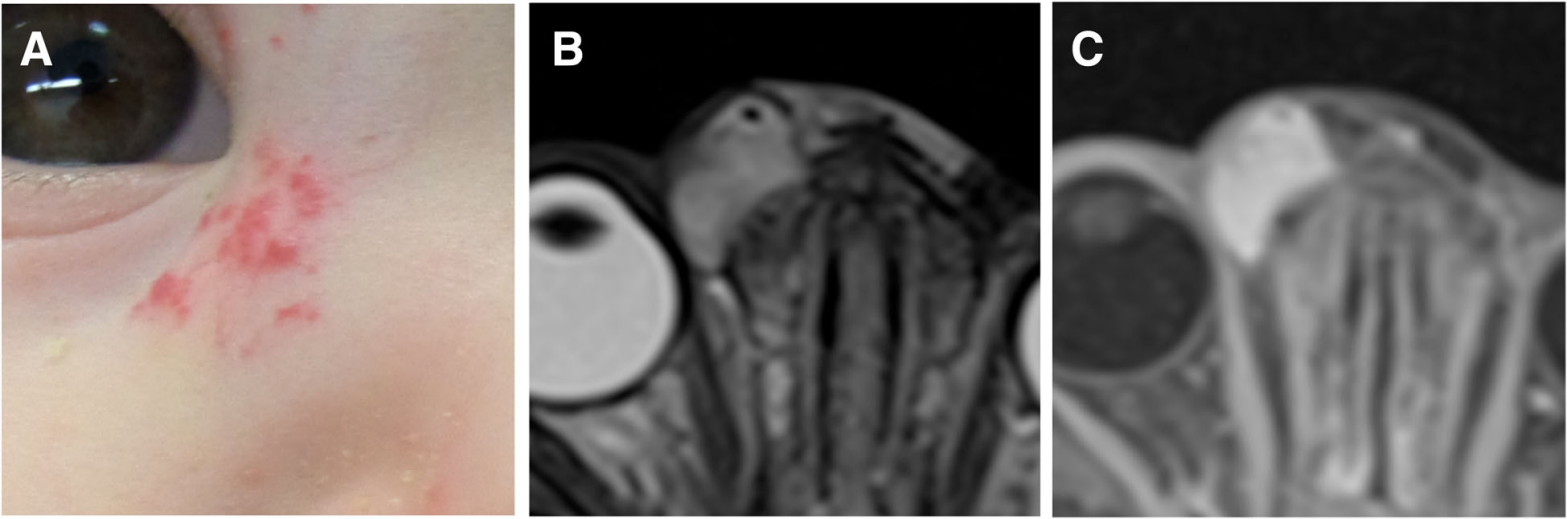
Infantile hemangioma in an 8-month-old boy. The tumor is superficially visible through disseminated red papules (**a**). On MRI, the tumor showed a typical homogeneous signal on T2w (**b**) and a corresponding homogeneous enhancement after Gadolinium administration (**c**)

**Fig. 2:**
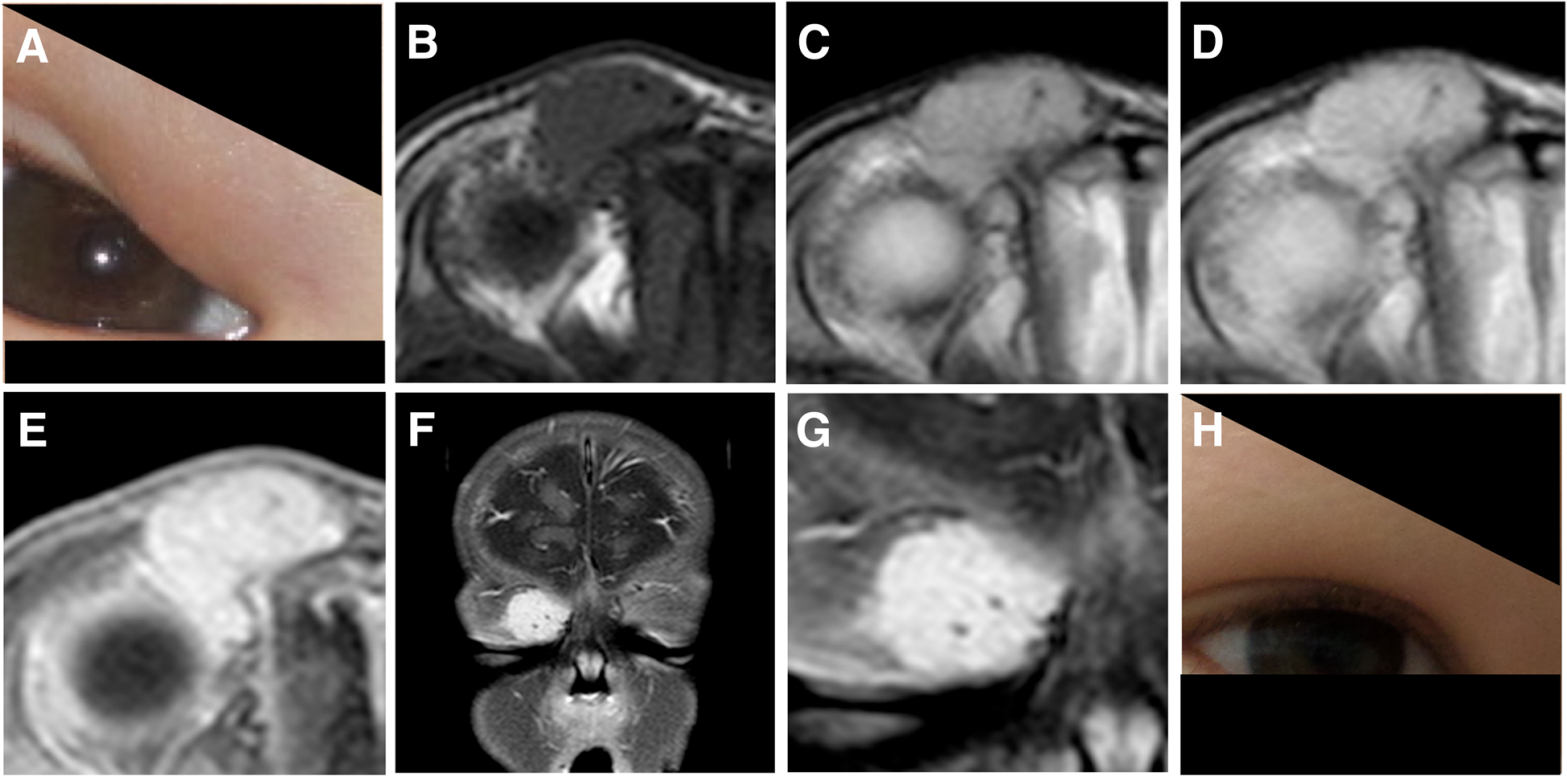
Infantile hemangioma in a 5-month-old boy invading the eyelid (**a**). The hemangioma exhibits a homogeneous architecture with only a few flow voids within the lesion (**b**–**d**). Similarly, a homogeneous contrast enhancement is visible after gadolinium administration (**e**–**g**). Two years later, the hemangioma involuted completely without any residual mass (**h**)

Precursor lesions can either be present at birth or occur later during the early neonatal weeks, either as areas of paleness due to local vasoconstriction or teleangiectatic red macules. Typically, infantile hemangiomas start to proliferate after a silent period of 1–3 weeks and appear as minimally elevated red papules or nodules (Fig. 1). Subsequent growth of hemangiomas is not linear but frequently completed by the age of 3–6 months [20]. After a period of relative stability, infantile hemangiomas regress (usually starting with the superficial components while deeper parts still proliferate, Fig. 2) in the following years with regression being completed in 90% of cases at the age of 4 years [21].

##### Diagnosis and radiological features

Most importantly, the diagnosis of infantile hemangiomas is a clinical one based on the raspberry-red cutaneous stains of increasing size with a sharp margin, usually not requiring either cross-sectional imaging or biopsy. Only in cases of difficult anatomical locations, diagnostic uncertainty, or associated complications, imaging is indicated. Hemangiomas typically are well-defined solid lesions both on ultrasound (US) as well as on magnetic resonance imaging (MRI). Depending on the composition of soft tissue versus vascular structures as part of their natural development cycle their echogenicity can vary being more hypoechogenic during progression compared to an increasing echogenic component (fatty tissue displacement of vessels) during regression. Doppler US can reveal both arterial and venous waveforms with high diastolic velocity, a high vessel density, and a Doppler shift > 2 kHz. On MRI, infantile hemangiomas are iso-to-intermediate intense on T1w, and intermediate-to-bright on T2w and PDw sequences (Figs. 1 and 2) and present as solid homogenous masses with sharp margins [22]. Internal flow voids can be present but are not as prominent as in arteriovenous malformations. Fat suppression techniques should be applied to as especially subcutaneous fat can mask or hide vast portions of the hemangioma. Of note, vascular malformations, both venous and lymphatic malformations, can be similarly bright on fat-saturated T2-weighted images and can thus be confused with hemangiomas. However, hemangiomas typically show a vigorous and rather homogenous enhancement after contrast agent administration, while vascular malformation need time to fill. Instead, lymphatic malformations only show a rim enhancement of the cystic lesions without central enhancement. As depicted in Figs. 1 and 2, it shall be emphasized that hemangiomas rather occur as homogenous enhancing mass instead of diffuse vascular structures, which is more common in vascular malformations.

##### Complications

**Fig. 3:**
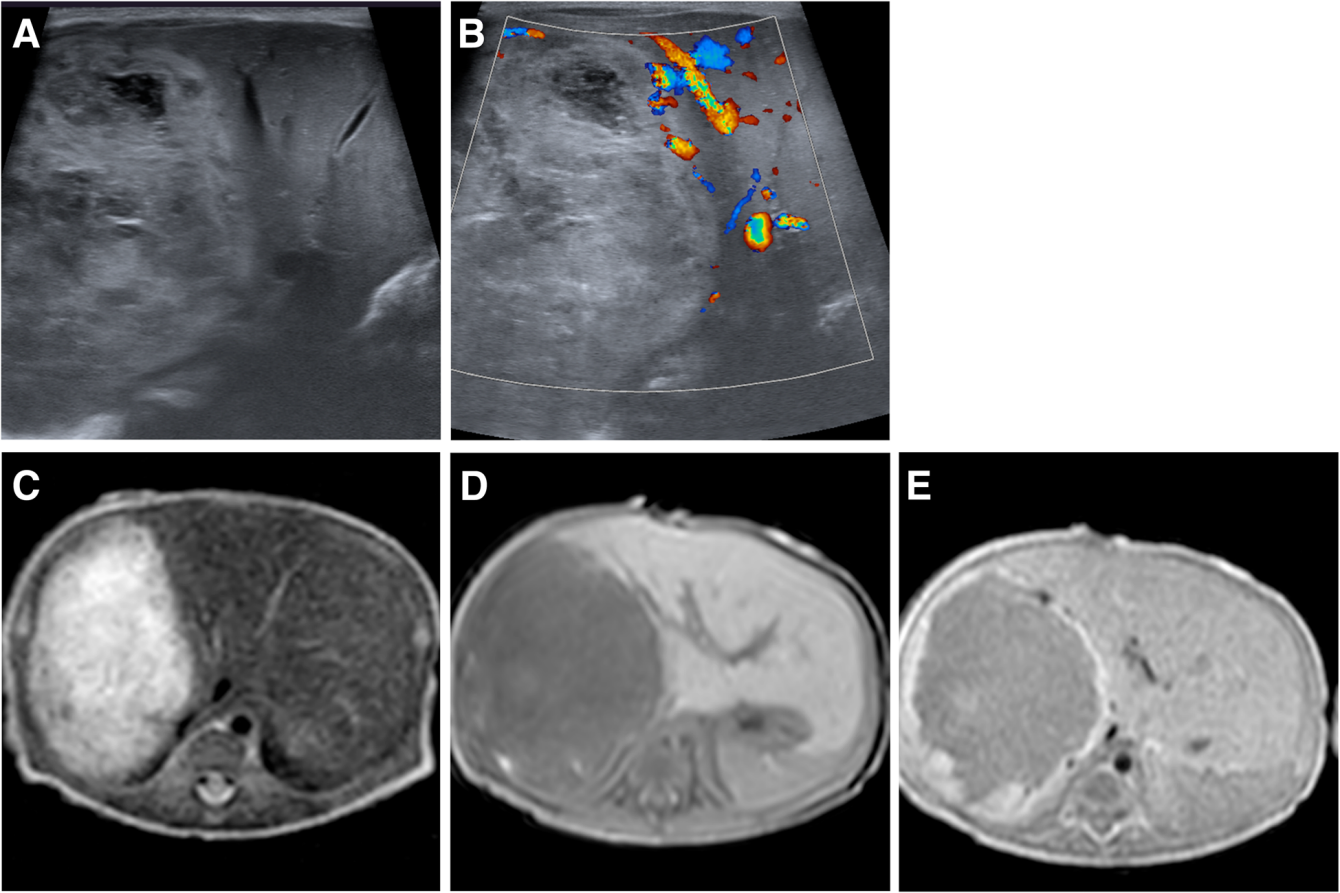
Rapid-involuting hemangioma (RICH) in a newborn. Ultrasound depicted a heterogeneous, fully developed mass invading large parts of the right liver lobe (**a**) with no flow-signal on Doppler ultrasound (**b**). MRI showed a rather homogeneous mass (**c**, **d**) with uniform enhancement after gadolinium administration (**e**). The tumor did not increase in size after birth and at the age of 8 months had decreases from 3.7 cm in maximum diameter at birth to 2.0 cm without the need for surgical or interventional treatment

Hemangiomas located close to the eyelid or bulb can lead to permanent amblyopia, astigmatism, or strabismus, in rare case incomplete eyelid closure and optic nerve injury can occur [23]. Ulceration leading to pain, bacterial superinfection, and discomfort occur in 10–25% of patients referred to dedicated vascular anomalies centers [24]. Disfigurement is especially critical in the centrofacial and parotid area, which tends to persist longer than elsewhere. Multifocal infantile hemangiomas, presenting disseminated as more than ten tumors, are frequently associated with visceral (typically hepatic) involvement, referred to as ‘neonatal hemangiomatosis,’ which can lead to high-output cardiac failure due to increased hemodynamics of the highly vascularized tumors [25]. Segmental infantile hemangiomas can be associated with various anomalies and result in complex syndromes such as PHACE (posterior fossa malformations, hemangiomas, arterial, cardiac, and eye anomalies) [9, 26]. As their name implies, segmental hemangiomas involve one or more segments of the face and trunk. This relates to the development of the fetus which takes place segmentally. Their involvement is thus more widespread. Segmental hemangiomas behave differently in that their growth pattern differs from common infantile hemangioma (Fig. 3).

##### Treatment

**Fig. 4:**
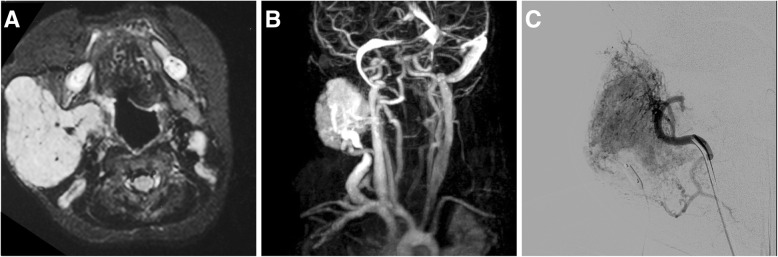
Embolization therapy in large infantile hemangioma with extensive perfusion. In MRI, the hemangioma presents as a rather homogeneous, solid mass with central flow voids (**a**). Dynamic MR-A shows massive AV shunting during the early venous contrast phase (**b**). Transarterial particle embolization was performed to reduce the flow and induce a regression with a protection balloon in the draining vein (**c**)

As infantile hemangiomas most frequently regress spontaneously, treatment is only indicated in complicated cases or tumor locations with increased morbidity and mortality (e.g., orbital cavity subglottic tumors, compression of neural structures, bleeding gastrointestinal tumors, large hemangiomas in danger of causing cardiac failure). Treatment of complicated infantile hemangiomas should always be performed within an interdisciplinary setting and include pharmacological therapy, surgery, or sometimes interventional approaches. Propranolol has emerged as the drug of choice for complicated infantile hemangiomas; the response rate is more than 98% with a dose of 2–3 mg/kg of body weight per day during a period of 6 months [27]. Besides its β-blocking potential, propranolol regulates cell proliferation in hemangiomas via catecholamines as well as the VEGF pathway [28]. Image-guided intralesional drug injections with bleomycin can cause severe local as well as systemic complications and are reserved only for exceptional cases. Surgical approaches, either open or laser, can be applied in the early course of disease either when propranolol is contraindicated or immediate treatment success is necessary to avoid complications (e.g. orbital or hepatic hemangiomas). However, surgery shall not be regarded as the standard treatment for infantile hemangioma. Laser surgery can be applied after the involutive phase to correct fibrofatty remnants or residual telangiectasia after the hemangioma has regressed in size and discolorization. Embolization therapy can be applied as a presurgical procedure to facilitate surgery and reduce perioperative bleeding rate in exceptional cases, but should be similarly not be considered as first-line therapy for infantile hemangiomas having the evident treatment results of propranolol in mind [29]. Transarterial embolization, preferentially being performed either small sized with particles (100–300 μm) or liquid embolics such as alcohol vinyl copolymer to penetrate deep into small arteries, has been reported to be safe and effective in infantile hemangiomas refractory to propranolol, high-output cardiac failure resulting from large shunt flow, intraoral and intranasal hemangiomas, hemangiomas with mass effect in difficult locations, symptomatic and ruptured hepatic congenital hemangiomas, and ulcerated hemangiomas with life-threatening bleeding [30–32] (Fig. 4).

#### Congenital hemangioma

Congenital hemangiomas are extremely rare; the exact prevalence however is unknown. They can resemble infantile hemangiomas both clinically as well as on imaging studies, but they are biologically a different entity. Clinically, they are recognized directly after delivery and appear rather bluish-red with less-well defined margins and may have a ‘pale-rim,’ as part of the adjacent skin is less well perfused due to a steal effect. Congenital hemangiomas are fully developed at birth (Fig. 3) and do not proliferate any more thereafter [10]. Typically, congenital hemangiomas are immunohistochemically negative for GLUT-1 in comparison to infantile hemangiomas. RICH are at or beyond their maximum extension at birth and regress rapidly in the months thereafter; regression is frequently complete with the first year of life [9, 33]. NICH instead persist without signs of regression, while rapid involution in PICH can occur and stop at any time, transforming PICH into NICH. Congenital hemangiomas, especially due to their extension already at birth, can cause significant complications such as high-output cardiac failure or thrombocytopenia, requiring early therapy.

#### Other hemangiomas

Spindle cell and epitheloid hemangiomas are benign vascular tumors, typically lesions involving the skin and subcutis only and mostly do not require radiologic imaging [34]. In rare cases, epitheloid hemangioma can develop as a reactive process following local trauma or pregnancy within the bone, presenting as lytic, well-circumscribed lesions in the metaphysis and diaphysis of long bones of the extremities [35, 36]. In such cases, lesions should be differentiated from intraosseous epitheloid hemangioendothelioma, which requires biopsy. There is no standard of therapy established and treatment may involve surgical curettage, complete resection, or sclerotherapy [34].

#### Pyogenic granuloma

**Fig. 5:**
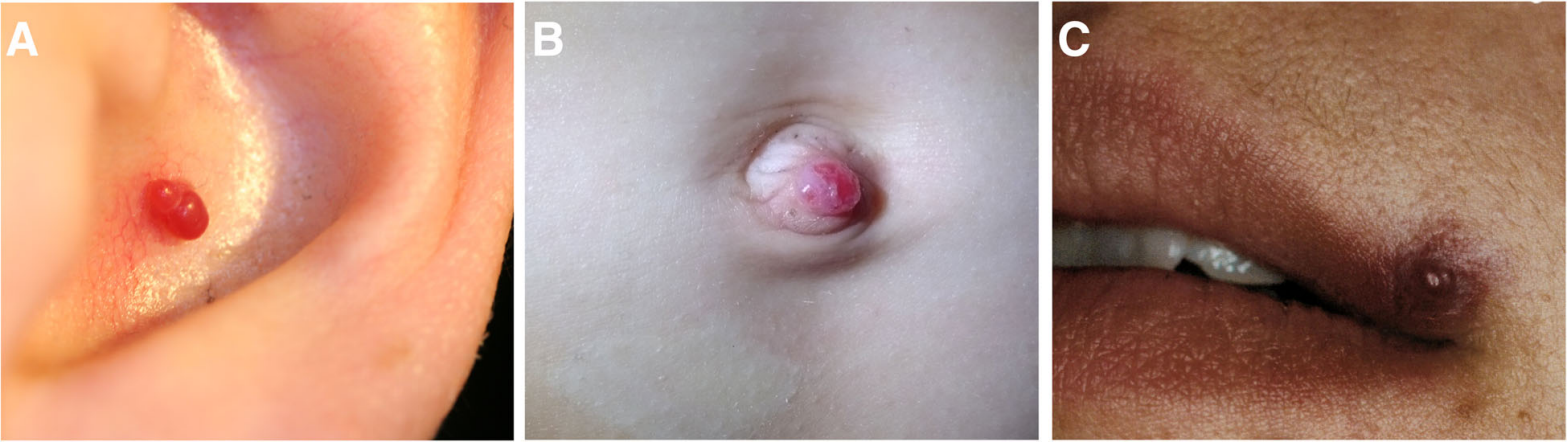
Various presentations of a pyogenic granuloma in the auricle (**a**), at the umbilicus (**b**) and at the lip (**c**)

Pyogenic granuloma can be a potential differential diagnosis of hemangioma. The prevalence is about 0.5–1% and thus represents a quite common acquired vascular tumor [37, 38]. Differing from infantile or congenital hemangioma, pyogenic granuloma rarely manifest before the age of 4 months. After minor injury, for example following insect bites, small vascular papules form up to 1 cm in size as red, exophytic lesions with high perfusion, primarily localized in the craniofacial region [9] (Fig. 5). However, pyogenic granuloma can also manifest within the intestines and parenchymal organs, which questions the formation out of minor injuries. Pyogenic granulomas, frequently also referred to as intravenous lobular capillary hemangioma, tend to bleed and are then best treated by surgical curettage or excision. Imaging is rarely indicated und thus no comprehensive data about presentation of pyogenic granuloma on US or MRI exists. Case reports describing MRI in pyogenic granuloma describe the morphology of pyogenic granuloma with those of hemangiomas, specifically an isointense signal of the lesion compared to the muscle and a homogenous enhancement after contrast agent administration [39]. Similarly, only rare evidence exists regarding interventional treatment of pyogenic granuloma, which may be reserved either for large tumors or for cases of severe hemorrhage [40, 41]. In general, although pyogenic granulomas can regress spontaneously, surgical excision is recommended to prevent bleeding complications and improve visible deformity.

### Locally aggressive or borderline vascular tumors

#### Tufted angioma and kaposifom hemangioendothelioma

**Fig. 6:**
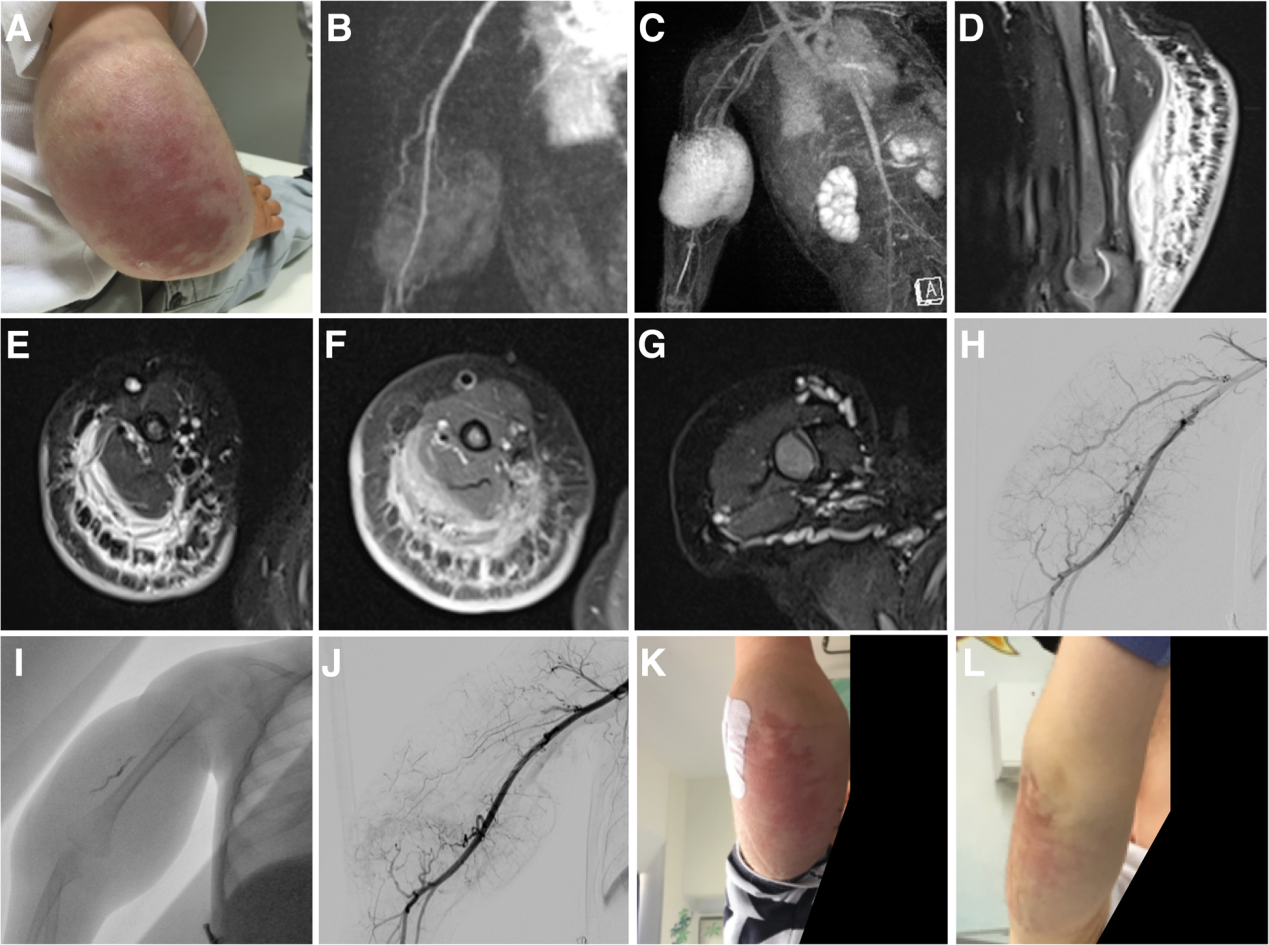
Kaposiform Hemangioendothelioma in a 13-month-old boy with moderate Kasabach-Merrit phenomenon (platelet count at 70,000/μl at admission). The boy presented with a large mass at the right upper arm, extending toward the elbow circumference of the distal humerus. The mass included severe purpura and ecchymoses, was tensely swollen with accompanying lymphedema (**a**). Arterial feeders to the lesion could be identified on MRA (**b**). A massive tumor blush was observed during the later arterial contrast phase (**c**). Fluid-sensitive sequences (**d**, **e**) revealed extended edema with characteristic septa perpendicular to the skin surface that showed contrast enhancement on delayed-phase T1w (**f**). Dilated veins were present around the primary tumor (**g**). Following an interdisciplinary consensus, it was decided to proceed with embolization, accompanied by treatment acetylsalicylate acid and sirolimus. Angiogram confirmed hypertrophic arterial feeders directed toward the lesion, but absent arterio-venous shunting (**h**). Onyx embolization was performed to exclude ~ 75% of the arterial tumor vasculature (**i**, **j**). Kasabach-Merrit phenomenon resolved with thrombocyte levels returned to normal 3 weeks after embolization. Three months after embolization and start of ASS and sirolimus treatment, the lesion had markedly regressed in size, the lymphedema had disappeared, and discoloration was regressing (**k**), 6 months later a mild discoloration persisted, without residual mass (**l**). Similarly, there was no relapse of the Kasabach-Merrit phenomenon

Rather than being two distinct entities, tufted angioma (TA) and kaposiform hemangioendothelioma (KHE) are nowadays understood as a spectrum of the same disease. Both are locally aggressive tumors that can be accompanied by life-threatening consumptive coagulopathy consisting specifically of severe thrombocytopenia and hyperfibrinogenemia with diffuse intravascular coagulation, referred to as Kasabach-Merritt phenomenon (KMP) [22, 34, 42]. In general, tufted angioma is seen as a more superficial and milder form of the borderline vascular tumor compared to KHE. Interestingly, both tumors present distinct lymphatic antigens which support the hypothesis that both entities have at least a partial lymphatic phenotype [42–44]. Of note, KHE and TA are negative for infantile hemangioma markers such as GLUT-1 [18, 42]. In case of diagnostic uncertainty, biopsy is recommended and histopathological evaluation is required from highly experienced pathologists with knowledge of these distinct pheno- and genotypes. The incidence of KHE is estimated to be below 1 in a million and most frequently KHE occur within the first weeks of life (60%), but can already be observed in utero or develop later in early adulthood [45]. KHE most frequently manifests along the extremities and less frequently along the trunk, the retroperitoneum, or the head-neck region. The clinical appearance is rather characteristic with a highly vascularized reddish-blueish mass without sharp margins. The skin is almost always involved with various degrees of purpura and ecchymoses (Fig. 6a). Larger KHE present as bulky mass with tense swelling of the affected extremity and frequently accompanied by massive lymphedema (Fig. 6a).

MRI is the imaging modality of choice to demonstrate disease extension [46, 47]. The mass is poorly circumscribed and diffusely permeates soft tissues without reference to defined tissue planes with potential destruction of adjacent bones. The lesions are highly vascularized and frequently hypertrophic arterial feeders can be identified on MRA (Fig. 6b). In the early phase, a massive arterial tumor blush occurs around the center of the lesion (Fig. 6b, c). Flow-voids can be observed, in contrary KHE never exhibit arterio-venous shunting which is a common feature of arteriovenous malformations. Imaging depicts the associated lymphedema with characteristic septa perpendicular to the skin surface (Fig. 6d, e), that show prominent enhancement after gadolinium administration (Fig. 6f). Dilated draining veins are frequently observed (Fig. 6g). The diagnosis is made clinically together with MR imaging and, most importantly, laboratory values. As > 70% of KHE develop a KMP, where activated metaplastic tumor endothelium activates thrombocytes and destroying them within the lesion, thereby causing massive thrombocytopenia and life-threatening consumptive coagulopathy [11].

Treatment of KHE, especially with associated KMP is complex and always needs dedicated specialized care within an interdisciplinary setting. In 2013, a consensus-derived standard of practices was published. In this report, intravenous treatment with vincristine and prednisolone has been advocated. Embolization therapy was suggested as a bridging therapy using either small-sized particles or liquid embolics, especially in life-threatening situations, providing additional time for medical therapy to become effective [34, 48]. Surgery may be performed but requires extensive tumor resection, is often incomplete or impossible due to the accompanying coagulation disorder, and is associated with substantial morbidity. A recent meta-analysis evaluated the treatment of 244 patients with KHE, which consisted of systemic corticosteroids, interferon, radiotherapy, embolization, aspirin/ticlopidin for inhibiting thrombocyte aggregation, chemotherapy, and sirolimus. Up to now, there is no consensus on the optimal therapeutic regime for KHE with or without KMP but several novel approaches are being discussed including the use of mTOR inhibitors [11]. The authors of this review have encouraging clinical results in a combined interdisciplinary treatment of KHE with life-threatening KMP. We propose to immediately initiate treatment with acetylsalicylate acid in a weight-adapted dose of 1–2 mg per kg/body weight. Subsequently, we perform transarterial embolization using alcohol vinyl copolymer (Fig. 6h–j) to reduce the contact of thrombocytes to the activated endothelium and rescue the patient form the consumptive coagulopathy. Following embolization, we initiate treatment with sirolimus, a well-established mTOR inhibitor, which has proven effective in the treatment of vascular malformations with a high proliferation rate and has similarly been used in treatment of KHE [48–50]. We start sirolimus with a dose of 0.8 mg/m^2^ with the goal to achieve a trough level of 7–9 ng/ml in the early morning. In the cases we have treated so far, KMP resolved within the first 2 weeks of treatment eliminating the risk of life-threatening bleeding, and with the combination of embolization and sirolimus substantial regression of the size of the mass, reduction of discoloration and skin tension was achieved (Fig. 6k, l). Medical treatment with acetylsalicylate acid and sirolimus is continued for 6–12 months. Again, we would like to emphasize that especially in cases of KHE and KMP, tight cooperation between radiologists, pediatric hematooncologists, pediatric surgeons, hemostasiologists, and pathologists is absolutely mandatory, also with respect to the fact that all therapy approaches discussed are being performed as off-label use in the absence of published clinical data in this extremely rare tumor entity.

#### Other hemangioendotheliomas

Retiform hemangioendothelioma is a superficially located tumor that is frequently encountered in adults, less frequent in small children, and has been named due to its net-like vascular composition [51]. Retiform hemangioendothelioma are closely related to PILA/Dapka tumors and have an overlapping pathological background. Composite hemangioendothelioma is another rare vascular tumor entity, similar as retiform hemangioendothelioma with almost no malignant potential or tendency to metastasize [52]. Imaging appearance is heterogeneous [53] so that diagnosis has to be based regularly on biopsy.

#### Kaposi sarcoma

Kaposi sarcoma (KS) is considered a low-grade malignant vascular tumor. Four different subtypes are classified: classical, iatrogenic, AIDS-related, and African/endemic [42], while all subtypes share human herpesvirus 8 infection as their common origin. Infection of vascular endothelial cells by the HHV8 virus induces their lymphatic reprogramming and similar downregulation of classical vascular genes. Cutaneous manifestations are heterogeneous such as dermal patches, plaques, or nodules. Compared to adults, cervical and oropharyngeal lymphadenopathy as well as visceral involvement are more common in children than cutaneous manifestations. All tissues and organs can be affected, leading to a wide variety of presentations on radiological imaging best reviewed by O’Mahony [54] and Restrepo [55]. Mass-like lesions in deeper tissue show marked contrast enhancement and can even show high FDG uptake in PET/CT imaging. Lesions can result in progressive bone destruction and metastasis-like osteolytic lesions [56, 57]. Lymphatic manifestation over the entire body as well as characteristic perihilar bronchovascular infiltrates of KS are frequently encountered in imaging of KS. While iatrogenic type of Kaposi sarcoma frequently regresses spontaneously after ending immunosuppression, the other types require systemic therapy or/and local radiotherapy. Topical treatments such as cryotherapy or intralesional injection of Bleomycin or interferon have been reported.

### Malignant vascular tumors

#### Epithelioid hemangioendothelioma

**Fig. 7:**
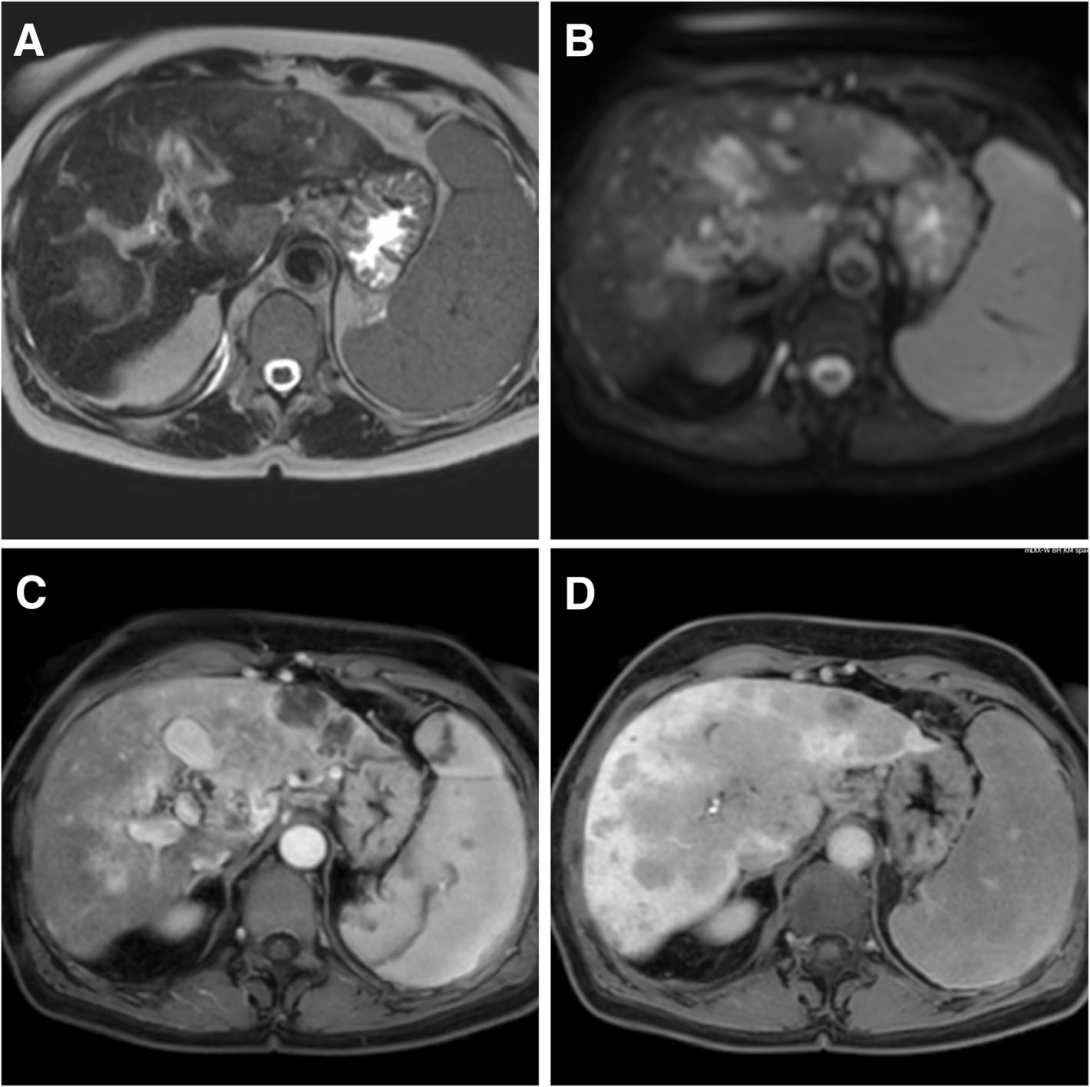
Epithelioid hemangioendothelioma of the liver in a 18-year-old woman. MR images depict diffuse spread of typical EHE tumor nodules over the entire liver. T2w (**a**), diffusion-weighted imaging (**b**), Dixon fat-water imaging in the early (**c**), and late phase (**d**) after administration of gadoxetic acid. Due to accompanying liver cirrhosis (child B), the patient rapidly developed progressive liver failure and could not undergo surgery or systemic therapy

Epithelioid hemangioendothelioma (EHE) in contrast to the aforementioned hemangioendotheliomas is a malignant tumor arising from the endothelium of medium to large blood vessels and can occur in the soft tissue of the extremities (rarely head and neck) as well as in liver, spleen, lung, and bone and lymphatic tissue [34]. The peak incidence (< 1/1 million) is in the fourth and fifth decade, but it can rarely occur also in children. Females under hormonal contraception seem to be affected more frequently as far as hepatic manifestations are concerned [58]. The degree of malignancy can vary and the overall survival has been reported to be 73% at 5 years. Frequently, EHE of the liver follows an indolent course, causing a continuous debate of immediate treatment is necessary or of benefit for the patient. There is no established tumor marker, and imaging findings are similarly heterogeneous, with moderate enhancement after contrast agent administration [59]. An example of hepatic EHE is shown in Fig. 7. Surgery is considered the treatment of choice for solitary lesions [60]. Once metastasized, the response to (angiosarcoma like) chemotherapy is poor [61]. Preoperative embolization has been reported either preoperatively or in cases of rupture [62, 63], chemoembolization either in case of inoperability or as bridge to liver transplant, with no clear evidence until today [64, 65].

#### Angiosarcoma

Angiosarcomas are similarly rare (estimated incidence 2/1 Mio) and comprise 2% of all sarcomas. In general, they are tumors of high malignancy that can arise from vascular endothelial cells in any part of the body. Typically, angiosarcomas manifest in the sixth or seventh decade of life, rarely they can occur at childhood age [66]. Multiple risk factors are known, such as chronic lymphedema (Stewart-Treves syndrome), conditions post-surgery and radiotherapy, various exogenous toxins such as arsenic or vinyl chloride, as well as genetic predisposition such as neurofibromatosis NF-1, BRCA1/2 mutations, Mafucci syndrome, as well as Klippel-Trenaunay syndrome, the latter two being predominantly combined vascular malformations. Although most angiosarcomas occur spontaneously, a few reports of malignant transformation of benign vascular lesions exist [67]. Cutaneous angiosarcomas may present as local bruise or papule without sharp borders, typically occur multifocal and can be easily mistaken for benign vascular lesions. With increasing size, the infiltrating growth character becomes more obvious. Soft tissue or visceral organ manifestations present as expanding heterogeneous mass, which can grow rapidly up to 20 cm or more in size [66]. Metastasis typically occurs hematogeneously most frequently to the lungs. Both cutaneous lesions as well as visceral manifestations exhibit wide possible differential diagnosis and similarly hepatic manifestation of angiosarcoma is radiologically difficult to differentiate from other liver tumors such as hepatocellular carcinoma, liver metastasis, or even hemangioma [68], so that biopsy of suspect lesions is required for correct diagnosis.

**Fig. 8:**
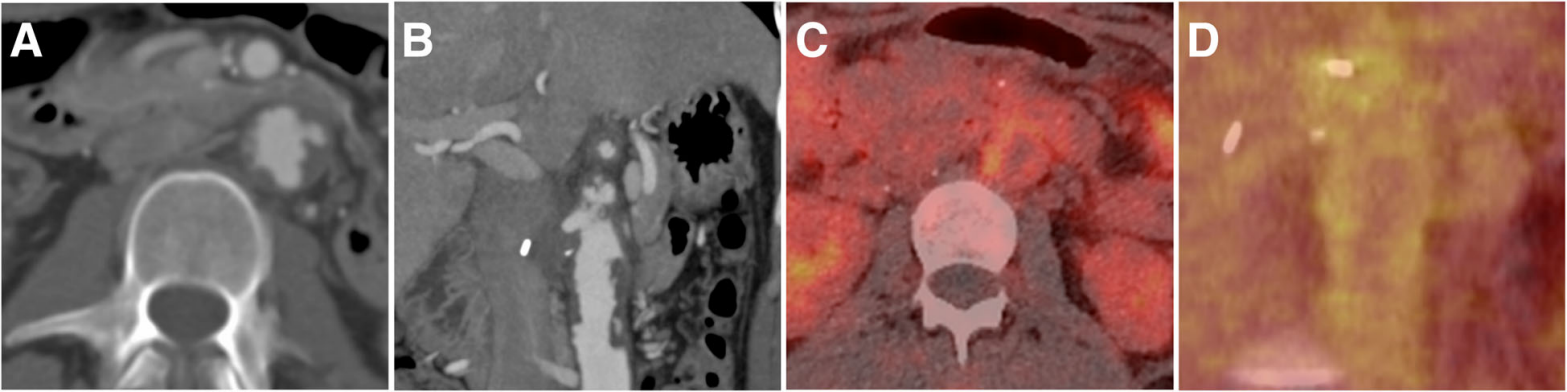
Angiosarcoma of the abdominal aorta in a 22-year-old woman. Computed tomography angiography (CTA) depicts tumorous mass extending within the aortic lumen resembling thrombus or atheroma (**a**, **b**). The intraluminal mass however showed increased metabolic activity on ^18^F- FDG-PET/CT (**c**, **d**), indicative of a malignant tumor

Imaging of primary lesions is most frequently performed by MRI or CT; CT of the chest is necessary to rule out pulmonary metastasis. In case of angiosarcoma of the large vessel, the tumor can mimic atheroma or thrombotic material within the vessel [69] (Fig. 8a, b). In such cases, PET imaging can help in the differential diagnosis, as angiosarcomas typically exhibit a high metabolic activity (Fig. 8c, d). Treatment of vascular complications includes palliative approaches in case of vessel obstruction or thromboembolic complications [70] as well as emergency embolization in case of tumor bleeding. For hepatic manifestations, chemo- and radioembolization have been performed in cases of inoperability or metastasis, but their efficacy compared to chemotherapy has not been evaluated [68, 71, 72].

#### Other

Besides the described tumors which are of endothelial origin, other vascular soft tissue tumors exist. Hemangiopericytoma is a distinct tumor entity arising from vascular pericytes. Several categories such as solitary fibrous tumors have been subcategorized as hemangiopericytoma-like neoplasms. Typically, hemangiopericytomas in childhood present as self-limiting lesions with malignant potential while tumor manifestation in adulthood is frequently malignant with high likelihood of metastasis, most commonly occurring in case of intracranial hemangiopericytomas. Hemangiopericytomas are hypervascular lesions. Treatment of these vascular soft tissue tumors is not standardized and involves surgery and/or radiosurgery for intracranial lesions in combination with systemic therapy. In rare cases, embolization can be performed preoperatively to reduce bleeding complications [73–76].

## Summary

Benign vascular tumors are frequent in children and are often misdiagnosed as vascular malformations and vice versa. This distinction is not a semantic one, but the treatment and natural course of a hemangioma is substantially different from vascular malformations. Besides benign vascular tumors, locally aggressive semi-malignant tumors exist which are rare but can present with life-threatening consumptive coagulopathies. Malignant vascular tumors are rare in children and are more frequent in adults. It is important to distinguish vascular neoplasm from other entities and observe the individual clinical picture. Radiological imaging, especially MRI, is not necessary for uncomplicated hemangiomas, where ultrasound is sufficient, but should be performed in every case with uncertain diagnosis. In cases with expected potential malignant potential, biopsy is required to define the vascular pheno- and genotype. Genetic testing is becoming increasingly important, both to define the appropriate diagnosis as well to better understand how these factors cause disruption of the vascular bed and give rise to dysplasia or metaplasia of human vascular cells [77]. Minimal-invasive approaches are becoming an important part of the interdisciplinary therapy regimens especially of pediatric vascular neoplasms and require dedicated skills from radiologists in the management of patients with vascular anomalies.
